# The Epidemiologic Comparison of Two Correlated Relative Risks: A Simple but Efficient Clinical Trial Design for Assessing Risk-Reduction and Treatment Significance

**DOI:** 10.3390/medicina62010070

**Published:** 2025-12-29

**Authors:** Jimmy T. Efird, Genevieve N. Dupuis, Yuk Ming Choi, Hongsheng Wu

**Affiliations:** 1 VA Cooperative Studies Program Coordinating Center, 2 Avenue de Lafayette, Boston, MA 02111, USA; gdupuis@bu.edu (G.N.D.); wuh@bu.edu (H.W.); 2 Department of Radiation Oncology, School of Medicine, Case Western Reserve University, Cleveland, OH 44206, USA; 3 Department of Biostatistics, Boston University School of Public Health, Boston, MA 02118, USA; 4 Provider Services, Signify Health, 4055 (S-700) Valley View Ln, Dallas, TX 75244, USA; ychoi@signifyhealth.com; 5 Division of Mathematics, Analytics, Science, and Technology, Babson College, Wellesley, MA 02457, USA

**Keywords:** common referent-control, correlated relative risks, log-difference scale, risk-reduction, umbrella trials

## Abstract

In the context of platform design, umbrella trials are a type of master protocol in which multiple treatments are randomized and evaluated with respect to a common, referent-control arm. In a simplified (1:1:1), non-adapted case, this is equivalent to the epidemiologic comparison of two correlated relative risks for assessing risk-reduction on the log-difference scale. The use of shared controls has the potential to reduce study time and costs but infers greater complexity to account for the covariance between relative effect estimates (i.e., dependence arising because treatment arms use the same referent group). Multiplicity adjustment is unnecessary on the log-difference scale (three-group design) as this is a single test statistic for interaction. An intuitive, risk-reduction (LDL-C statin) example is presented to illustrate the practical application of this method.

## 1. Introduction

An efficient and cost-effective clinical trial design involves comparing two or more treatment groups to a single, shared referent-control arm. This reduces the overall number of patients randomized to a comparison group by half, offering greater appeal to patients, drug companies, and investors [1]. In the basic instance of a new versus legacy therapy, relative to a common control group, this epidemiological approach is innovative, practical, and methodologically rigorous. A key advantage of the (1:1:1) design entails the simultaneous assessment of risk-reduction and treatment significance.

Parallel drug comparisons of this type are known as master protocol, platform studies, with the term “umbrella trial” denoting the assignment of patients to one of many therapies, typically based on their molecular characteristics [2]. When aptly applied, this strategy has clear and direct implications for real-world, clinical trial practice, culminating in reduced study time, costs, and bias.

The shared reference group usually consists of a mutually distinct, random collection of patients, but it can also be assembled from pooled controls of previously conducted clinical trials, historic collections, or registries. In the case of an observational, targeted trial emulation, controls need to meet the eligibility criteria at the time of case definition [3]. Regardless of study type, the sharing of controls is appropriate only if treatment arms are comparable with respect to the referent base population in terms of key attributes (e.g., time period, geographic location, and demographic makeup).

An important feature of this design is the correlated structure of the data, requiring the incorporation of a covariance term into the denominator of the test statistic. The need to account for multiplicity adjustment is offset in the simple, three-group (risk-reduction) design, as the analysis is based on a single log-difference test for interaction [4]. A generalized linear model (GLM) with a log-link function may be used to analyze the correlated data, which can accommodate both univariable and adjusted models for risk-reduction [5].

## 2. Preliminaries

### 2.1. Definition of Two Correlated Relative Risks in Terms of a 3 × 2 Cross-Tabulation Table

Let $\left(a_{ij}\right)$ denote the $\left({i^{th},j}^{th}\right)$ cell sizes for a contingency table with i=1 to 3  rows and j=1 to 2 columns (see Table 1). Here, the rows represent the treatment exposures $\left(T_{0},T_{1}{,T}_{2}\right)\;$ while the columns correspond to an unfavorable vs. favorable outcome in the context of risk-reduction. Define the two relative risk (RR) estimates being compared with respect to a common referent-control group $\;\left(T_{0}\right)\;$ as follows:

(1) RR1^=a11a11+a12a31a31+a32=a11a1+a31a3+=π^0π^2

and

(2) RR2^=a11a11+a12a21a21+a22=a11a1+a21a2+=π^0π^1,

where $\left(a_{1,1}\right),\left(a_{2,1}\right),\;and\left(a_{3,1}\right)\;$ are binomially distributed variables. Accordingly, $\left({\hat{RR}}_{1}\right)$ represents the ratio of the event probability under $\left(T_{0}\right)$ to $\left(T_{2}\right)$, while $\left({\hat{RR}}_{2}\right)$ compares $\left(T_{0}\right)$ to $\left(T_{1}\right)$.

### 2.2. Test Statistic and p-Value

Consider the following sample test statistic $\left({\hat{\zeta }}_{RRR}\right)$ for comparing the ratio of two correlated RR estimates on the log-difference scale (i.e., $\left[log\left({\hat{RR}}_{1}\right)-log\left({\hat{RR}}_{2}\right)\right]=log\left(\hat{RRR}\right)$), with respect to a common referent-control group:

(3) $${\hat{\zeta }}_{\mathrm{RRR}}=\frac{log\left(\frac{{\hat{RR}}_{1}}{{\hat{RR}}_{2}}\right)-E\left[log\left(\frac{{\hat{RR}}_{1}}{{\hat{RR}}_{2}}\right)\right]}{\sqrt{Var\left[log\left({\hat{RR}}_{1}\right)\right]+Var\left[log\left({\hat{RR}}_{2}\right)\right]-2Cov\left[log\left({\hat{RR}}_{1}\right),log\left({\hat{RR}}_{2}\right)\right]}}\;,$$

where the denominator estimates the square-root of the variance for $log\left(\frac{{\hat{RR}}_{1}}{{\hat{RR}}_{2}}\right).$ $E\left(*\right),Var\left(*\right)$ $and\;Cov\left(*\right)$ denote the expectation, variance and covariance of the quantities indicated within parenthesis. The expectation of the log-difference, i.e., $E\left[log\left(\frac{{\hat{RR}}_{1}}{{\hat{RR}}_{2}}\right)\right]$, equals zero under the null hypothesis (H_0_) that the two RRs are equivalent (i.e., ${RR}_{1}={RR}_{2}$), versus the alternative (H_1_) that they differ (i.e., ${RR}_{1}\neq {RR}_{2}$).

When the sample sizes for each group $\left(n_{{\hat{RR}}_{1}},n_{{\hat{RR}}_{2}}\right)$ are sufficiently large, $\left({\hat{RR}}_{1},\;{\hat{RR}}_{2}\right)$ will tend to be conditionally independent, and one can simply estimate the denominator of $\left({\hat{\zeta }}_{RRR}\right)$ by $\sqrt{{\left\{SE\left[log\left({\hat{RR}}_{1}\right)\right]\right\}}^{2}+{\left\{SE\left[log\left({\hat{RR}}_{2}\right)\right]\right\}}^{2}\;}$, where $SE\left(*\right)$ is defined as the standard error. That is, as $\left(n_{{\hat{RR}}_{1}},n_{{\hat{RR}}_{2}}\right)\to \infty ,$ sample estimates become more reliable and stable, with the covariance term approaching zero and $\left({\hat{\zeta }}_{RRR}\right)$ assuming a standard normal distribution. However, for smaller sample sizes, the relative effect estimates are less accurate and more prone to random fluctuation (variability). To warrant approximate normality of the test statistic for reasonably sized, non-asymptotic samples, it remains integral to account for the sample covariance term in the denominator.

The corresponding *p*-value for interaction $\left(P_{Int}\right)\;$ is computed as follows:

(4) $$P_{Int}\sim 2\cdot \left\{1-\Phi \left(\hat{\zeta }\right)\right\},$$

where

(5) Φζ^=∫−∞ζ^RRRe−x222·πdx.

### 2.3. Analytic Details

#### 2.3.1. Delta Approximation

The “Delta (δ) method” provides a first-order Taylor series approximation for the variance of a function involving random variables with known, finite moments [6]. By defining $\left(y\right)$ as the function of two variables $\left(x_{1}\;{and\;x}_{2}\right)$, each with a small coefficient of variation, it follows that

(6) $$Var\left(y\right)≅{\left(\frac{\partial y}{\partial x_{1}}\right)}^{2}Var\left(x_{1}\right)+2\left(\frac{\partial y}{\partial x_{1}}\right)\left(\frac{\partial y}{\partial x_{2}}\right)Cov\left(x_{1},x_{2}\right)+{\left(\frac{\partial y}{\partial x_{2}}\right)}^{2}Var\left(x_{2}\right),$$

where the partial derivatives of $\left(y\right)$ with respect to $\left(x_{1}\;and\;x_{2}\right)$ are evaluated at their respective mean values. The method assumes a local linear approximation of the underlying response surface, with optimal results realized when the function assumes an asymptotically Gaussian distribution.

#### 2.3.2. Sample Variance and Covariance Estimates

Applying the δ-method, it is easily seen that

(7) $$Var\left[log\left(\hat{{RR}_{1}}\right)\right]≅{\left(\frac{a_{1+}}{a_{11}}\right)}^{2}\left\{\frac{\frac{a_{11}}{a_{1+}}\left(1-\frac{a_{11}}{a_{1+}}\right)}{a_{1+}}\right\}+{\left(\frac{a_{3+}}{a_{31}}\right)}^{2}\left\{\frac{\frac{a_{31}}{a_{3+}}\left(1-\frac{a_{31}}{a_{3+}}\right)}{a_{3+}}\right\}$$

(8) $$≅\frac{1}{a_{11}}-\frac{1}{a_{1+}}+\frac{1}{a_{31}}-\frac{1}{a_{3+}}.$$

Accordingly,

(9) $$SE\left[log\left(\hat{{RR}_{1}}\right)\right]\;\;≅\sqrt{\frac{1}{a_{11}}-\frac{1}{a_{1+}}+\frac{1}{a_{31}}-\frac{1}{a_{3+}}}.$$

Likewise,

(10) $$SE\left[log\left(\hat{{RR}_{2}}\right)\right]\;≅\sqrt{\frac{1}{a_{11}}-\frac{1}{a_{1+}}+\frac{1}{a_{21}}-\frac{1}{a_{2+}}}.$$

As the only common term for $\left({\hat{RR}}_{1},\;{\hat{RR}}_{2}\right)$ is π^0=a11a1+, it readily follows from the δ-method that

(11) $$Cov\left[log\left(\hat{{RR}_{1}}\right),log\left(\hat{{RR}_{2}}\right)\right]\;≅\;\frac{Cov\left(\hat{{RR}_{1}},\hat{{RR}_{2}}\right)}{{\hat{\pi }}_{0}^{2}}$$

(12) $$≅\frac{Var\left({\hat{\pi }}_{0}\right)}{{\hat{\pi }}_{0}^{2}}$$

(13) $$≅\frac{{\hat{\pi }}_{0}\left(1-{\hat{\pi }}_{0}\right)}{\pi_{0}^{2}a_{1+}}$$

(14) $$≅\frac{\left(1-\frac{a_{11}}{a_{1+}}\right)}{a_{11}}$$

(15) $$≅\left(\frac{1}{a_{11}}-\frac{1}{a_{1+}}\right)$$

This result reflects the variance component attributable to the shared, referent-control arm (i.e., the first two terms of $Var\left[log\left(\hat{{RR}_{1}}\right)\right]).$

### 2.4. Comparison of Two Correlated Odds Ratios

Rather than RRs, the relative effect measure of interest may be odds ratios (ORs). An important aspect of ORs is that the estimate is “invariant to rotation”, meaning that the disease and exposure ORs are equivalent. Importantly, ORs are a versatile measure of association, whether analyzing incidence-density or cumulative-incidence studies. When the outcome is rare in both the exposed and unexposed groups, the RR and OR estimates are approximately equal. Independent of the rare disease assumption, ORs are also fairly accurate estimates for rate ratios when the proportion of the population exposed and disease incidence remain constant over time.

Analogous to the test statistic for correlated RR estimates, two ORs may be compared with respect to a common referent-control group on the log-difference scale. That is, with the covariance term implicit in the denominator,

(16) $${\hat{\zeta }}_{\mathrm{ROR}}=\frac{log\left({\hat{OR}}_{1}\right)-log\left({\hat{OR}}_{2}\right)}{\sqrt{Var\left[log\left(\frac{{\hat{OR}}_{1}}{{\hat{OR}}_{2}}\right)\right]}}\;,$$

where the subscript in ${\hat{\zeta }}_{ROR}\;$ denotes the log-ratio of ${\hat{OR}}_{1}$ and ${\hat{OR}}_{2}$. Again, using the 3 × 2 cross-tabulation table notation, the two sample ORs being compared with respect to a common referent-control group are defined as follows:

(17) OR1^=a11a12a31a32,

and

(18) OR2^=a11a12a21a22.

Applying the δ-method and rearranging, we have the following [7]:

(19) $$Var\left[log\left(\hat{{OR}_{1}}\right)\right]≅\frac{1}{a_{11}}+\frac{1}{a_{12}}+\frac{1}{a_{31}}+\frac{1}{a_{32}},$$

(20) $$Var\left[log\left(\hat{{OR}_{2}}\right)\right]≅\frac{1}{a_{11}}+\frac{1}{a_{12}}+\frac{1}{a_{21}}+\frac{1}{a_{22}},$$

and

(21) $$Cov\left[log\left(\hat{{OR}_{1}}\right),log\left(\hat{{OR}_{2}}\right)\right]\;≅\left(\frac{1}{a_{11}}+\frac{1}{a_{12}}\right).$$

### 2.5. Multinomial Distribution and Simulated Exact Statistics

Parameter estimates and the underlying distribution for $\left({\hat{\zeta }}_{RRR}\right)$ may be easily obtained by simulating observations from a multinomial distribution [8,9]. In effect, this provides exact statistics, which may be preferable when the sample sizes are very small and the normality of the test statistic is questionable. The simulated values are also useful for validating the large sample variance and covariance estimates obtained by the δ-method.

Conditioning on the total number of patients $\left(n\right)$, the probability that a mutually exclusive set of $\left(k\right)$ non-negative random variates $\left(Z_{1},Z_{2},\ldots ,Z_{k}\right)$ takes on a particular value ($z_{1},z_{2},\ldots ,z_{k}$) is given as follows:

(22) $$P_{n}\left(Z_{1}=z_{1},Z_{2}{=z}_{2},\ldots ,Z_{k}{=z}_{k}\right)=\frac{n!}{\prod_{i=1}^{k}z_{i}!}\prod_{i=1}^{k}{P\left(Z_{i}=z_{i}\right)}^{z_{i}},$$

where

(23) $$\sum_{i=1}^{k}P\left(Z_{i}=z_{i}\right)=1,$$

(24) $$\sum_{i=1}^{k}z_{i}=n,$$

and

(25) $$0<P\left(Z_{i}=z_{i}\right)<1.$$

The estimated probability for the $\left({i^{th},j}^{th}\right)$ cell of a 3 × 2 multinomial table for comparing two correlated relative effect estimates is given as $\left(\frac{a_{ij}}{n}\right)$.

## 3. Computational Methods

Analyses were performed and validated in SAS 9.4 (Cary, NC, USA). The GENMOD procedure for implementing GLMs was used to compute RRs (log-link function), ORs (logit-link function), and respective *p*-values for interaction. The interactive matrix language procedure (PROC IML) was used to simulate values from a multinomial distribution.

## 4. Relative Risk-Reduction Example

Consistently high blood levels of low-density lipoprotein (LDL-C) underlie a condition known as atherosclerotic cardiovascular disease (ASCVD), which manifests as the accumulation of plaque in the arteries (Table 1). Cardiologists recommend achieving LDL-C levels of less than 70 mg/dL following therapeutic intervention. A randomized clinical trial was undertaken to assess the benefit of a new (molecularly targeted) statin drug $\left(T_{2}\right)$ over a previously approved (standard) agent $\;\left(T_{1}\right)$, by way of a common referent-control arm of diet and exercise $\left(T_{0}\right)$. A 33% relative risk-reduction on the log-difference scale was observed following 18 months on the new statin therapy combined with diet and exercise (*P*_Int_ = 0.01994), versus standard treatment. In comparison, the estimated OR reduction on a log-difference scale was 167%, with a *p*-value for interaction of 0.01857, illustrating an exaggerated result for the ratio of ORs, owing to the frequent outcome event.

The manually obtained results provided in Table 1 are easily validated within rounding error against the PROC GENMOD output shown in Appendix A. For example,

(26) $$Var\left[log\left({\hat{RR}}_{1}\right)\right]=\left(0.1139^{2}\right)=0.01297,$$

(27) $$Var\left[log\left({\hat{RR}}_{2}\right)\right]=\left(0.0776^{2}\right)=0.00602,$$

(28) $$Cov\left[log\left(\hat{{RR}_{1}}\right),log\left(\hat{{RR}_{2}}\right)\right]=\frac{\left(0.01297+0.00602\right)-\left(0.1236^{2}\right)\;}{2}=0.00186$$

and,

(29) $$log\left(\frac{{\hat{RR}}_{1}}{{\hat{RR}}_{2}}\right)=log\left(\frac{1.5000}{1.1250}\right)=log\left(1.3333\right)=0.28768$$

## 5. Simulated Exact Results

**Figure 1:**
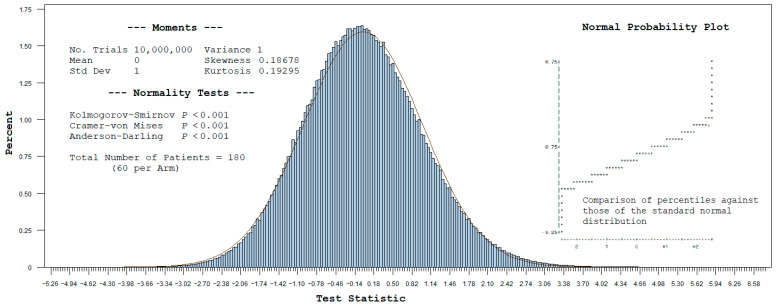
Histogram with standard normal distribution overlay (test statistic = ${\hat{\zeta }}_{RRR}$).

A multinomial distribution was used to obtain 10 million 3 × 2 tables based on the cell values in Table 1, with the frequency (histogram) plot for the test statistic shown in Figure 1. Given the relatively small sample size of 60 patients per arm, this is seen to follow a slightly skewed standard normal distribution (compared with solid Gaussian line), with non-zero skewness (0.18678) and kurtosis (0.19295). In this specific case, the distribution is right (positive)-skewed, wherein the tail is longer on the right side. Also note the higher peak of the simulated distribution, representing the value with the greatest probability.

The simulated exact results are given in Table 2. The area to the right of the test statistic under this distribution gives the simulated exact *p*-value for the interaction. Comparatively, the variance and covariance estimates obtained by the δ-method are reasonably close to the simulated exact statistics. Given the mild, right skewness of the simulated distribution, the *p*-value for interaction obtained by the exact method (i.e., 0.01566) is slightly more significant than that obtained by the normal theory method (i.e., 0.01994). However, this may not always be the situation for other examples, and one cannot assume that the normal theory result will universally yield the more conservative *p*-value.

## 6. Discussion

### 6.1. Overview

Master protocol platform studies embody an optimal approach for conducting parallel, clinical trials. Efficiencies are gained by using a shared control arm, which conveys a correlated, economical structure to the data. The manuscript at hand focuses on a non-adaptive (1:1:1) design. This simplified approach is equivalent to the assessment of two relative-risk estimates, with each epidemiologic measure being dependent on a collective denominator (single comparison group). When the disease outcome is rare among the two drugs being compared, a clinical trial may be emulated as a population-based, retrospective design, with a common referent group.

For moderately sized studies, as demonstrated with an example of n=180 patients (60 per arm), the (1:1:1) design is reasonably robust to departures from normality and can be easily analyzed as a GLM with a log-link function for RRs, which transforms the mean (µ) to the natural logarithm of (µ). This is in contrast with the logit-link function for ORs, which transforms the probability (µ) to the log-odds. The single, *p*-value for interaction offsets the need for multiplicity adjustment. Furthermore, the procedure is readily implemented using standard statistical software, with the option of including covariates to account for confounding.

In addition to clinical trials, the analysis of correlated data occurs in many epidemiologic settings (e.g., matched-pairs designs, pre- and post-studies, twin research, and cross-over investigations) [8]. A 2 × 2 × 2 three-way contingency design entailing the analysis of two paired binomial responses (measured on two treatments) has been used to compare side-effects of general anesthesia [10]. An overlapping collection of ~3000 controls has been implemented in a large genome-wide association study of ~2000 cases collected from seven diseases [11]. While methods to correct for a common referent-control group in association studies have been amply described in the literature, the emphasis has mainly been on correlated proportions and ORs [12,13]. In contrast, the current effort focuses on measuring the risk-reduction encountered when comparing two correlated RR estimates with a shared, dependent control arm. As a marginal method (versus ORs), the technique is appropriate to use for common diseases and prospective clinical analyses.

### 6.2. Advantages

A commonly employed clinical trial design involves directly comparing a new treatment against a standard agent and assessing the absolute effect risk-reduction (i.e., event rate difference between groups). However, the results may be biased because this design does not compare therapies with respect to a common, referent-control arm. For example, it may be challenging to prove the clinical usefulness of the new treatment if the standard drug was approved many years ago and is no longer as effective, owing to manufacturing changes, practice deviations, interactions with newer concomitant medications, and variations in the disease process. Assessing relative effect risk-reduction on a log-difference scale, using a shared referent group, effectively minimizes this threat to internal validity.

Another advantage of this design is accelerated drug development (i.e., shorter study time) and reduced resource allocation. Minimizing redundant control groups decreases the sample size of a study while increasing study power.

### 6.3. Limitations

A limitation of the normal theory approach is that the sample test statistic is reliant on the δ-method for obtaining variance and covariance estimates. This technique assumes that the first three partial derivatives of the underlying function are continuous, differentiable, and assume an asymptotic Gaussian form [14]. In most computer applications, only a first-order Taylor series approximation is used to derive variance estimates. Consequently, the asymptotic normality assumption may be questionable in small sample cases, with the conditional central limit theorem failing to hold true [15]. Increasing the sample size or using a second-order or higher Taylor series approximation may help to reduce bias. The exact method may need to be used if the data is particularly sparse or the rate of approaching normality is ostensibly slower than $O\left(1/\sqrt{n}\right).$ Barring extreme degenerate examples, the test statistic in practice typically assumes a moderately well-behaved, bell-shape distribution (for treatment groups of ~60 or more participants), and one may posit that the underlying data is sufficiently close in form to safely proceed with normal theory methods.

Under certain circumstances, convergence issues with the Bernoulli likelihood may occur when implementing the GLM approach with a log-link function. That is, the estimated probability of success vis-à-vis the Newton–Raphson algorithm may fall on or near the boundary of the parameter space (i.e., unity) [10]. One solution is to assume a Poisson likelihood and use the robust “generalized estimation equations method” to estimate the variance [16]. However, since Poisson regression allows predicted probabilities to exceed one, resulting confidence intervals will be slightly biased. An alternative workaround is to obtain estimates by applying the expectation–maximization (EM) algorithm [17]. The exact method based on re-parametrization of covariates is another promising approach. However, a rate-limiting aspect of the latter method is that the covariate vector of fitted probabilities equals unity and needs to be confirmed in advance [18].

The potential for misclassification bias may be a concern if a shared control is using either of the drugs under consideration at baseline, or begin their use after randomization (immortal time effect) [19]. Protocol deviations of this type must be carefully monitored during the course of the study and appropriately accounted for in the statistical analysis and reporting of results. Investigators should be vigilant of concomitant medications that may either intensify or diminish the referent effect. The selection of controls in a non-random fashion, or from a hospital source related to the outcome measure, poses another limitation that can be exacerbated with the use of a single-arm control group.

While reducing the time and cost of a study, combining external controls with randomized trial data can introduce complications, such as unmeasured confounding and collider bias. Chronological bias represents another concern, wherein aspects of the control group may change over time because of dependent temporal effects (e.g., practice changes, staff learning, unobserved time trends) [20]. Furthermore, the randomization of participants to the two treatment groups versus a common control arm “does not preclude confounding except for extremely large studies” [21].

Early termination or the censoring of participants poses a source of bias if differential in effect. Appropriate imputation methods suitable for correlated data may need to be implemented in such cases. State transition models or a counting process approach present other options for mitigating bias. When the effect is believed to be non-differential, the sample size may be increased in an adaptive fashion to offset bias toward the null. Deleting the affected participants from the analysis, if small in number, may be reasonable if appositely acknowledged.

The simplified (1:1:1) design for assessing risk difference on a log-difference scale does not require multiplicity adjustment when using shared controls. However, this may not be true for more complicated, multiple-arm, platform or umbrella trials that similarly utilize a common, referent arm. In general, it is best to consult a PhD-trained Epidemiologist or Statistician when designing a master protocol to ensure that the selected approach is valid for the application at hand and appropriately powered. This is especially important if one plans to use a flexible design that allows for adding or deleting treatment arms after trial commencement.

### 6.4. Future Directions

Future investigations are directed at innovative extensions of the log-difference, risk-reduction approach for conducting and analyzing clinical trials. This includes Bayesian hierarchical, mixed-model alternatives, and nonparametric adaptive methods, as well as efficient algorithms to compute conditional power. Additional consideration of designs using a shared attention-control arm would be informative. The latter would be explicitly designed to account for the bias of interacting with study staff, which is distinct from the compounds under study.

While the FDA recognizes the use of external controls in situations where conventional controls are not medically feasible or ethical (e.g., “rare conditions or indications lacking clinical equipoise for a concurrent control”), it remains imperative that the selection of nonconcurrent referents are representative of and generalizable to the targeted treatment population currently under study, as is true for all controls [22,23]. This includes “diseases with high and predictable mortality or signs and symptoms of predictable duration or severity”. The future development of novel techniques for adjusting nonconcurrent referents to the base population will be beneficial as such control groups become more commonly used in the log-difference, risk-reduction method at hand.

The selection of controls from an observational source can pose methodologic concerns as patients may “enter and exit the database at various times, ages, disease states, etc.” [23]. Additional research addressing this concern is needed.

## 7. Conclusions

The epidemiologic assessment of risk-reduction on the log-difference scale, using a shared referent-control arm, presents an efficient clinical trial design for comparing two correlated relative risk estimates of treatment effects. This method represents a simplified version of a multi-arm, parallel designed umbrella-platform trial without the need for multiplicity adjustment. The resulting *p*-value for the interaction is readily obtained using a GLM algorithm available in standard statistical software packages.

## Figures and Tables

**Table 1:** Clinical trial analysis (total number of patients = 180; 60 per arm).

Treatment (T)		Cholesterol Level (mg/dL) at 18 Months Post-Baseline		Results ^†^
		≥70 Unfavorable	<70 Favorable	
$T_{0}$ ^§^	Diet and Exercise	$a_{11}\;$ = 54	$a_{12}=\;$ 6	${\hat{RR}}_{1}\left(T_{0}:\;T_{2}\right)=1.5000$ ${\hat{RR}}_{2}\left(T_{0}:\;T_{1}\right)=1.125$0 $log\left(\frac{{\hat{RR}}_{1}}{{\hat{RR}}_{2}}\right)=log\left(1.3333\right)=\;0.28768$ $Var\left[log\left({\hat{RR}}_{1}\right)\right]$ = 0.01296 $Var\left[log\left({\hat{RR}}_{2}\right)\right]$ = 0.00602 $Cov\left[log\left(\hat{{RR}_{1}}\right),log\left(\hat{{RR}_{2}}\right)\right]\;$ = 0.00185 ${{\hat{\zeta }}_{RRR}=2.3275;\;\;P}_{Int}$ = 0.01994 ^¶^ RLD = $\left(\frac{{\hat{RR}}_{1}}{{\hat{RR}}_{2}}-1\right)\;\times \;100$ = 33.333%
$T_{1}$	Standard Statin + Diet and Exercise	$a_{21}=\;$48	$a_{22}=\;$ 12	
$T_{2}$	New Statin + Diet and Exercise	$a_{31}=\;$ 36	$a_{32}=\;$ 24	

^†^ Estimates rounded to 5 significant digits versus a fixed number of decimal places (Goldilocks method). ^§^ Common referent-control. Baseline = initiation of therapy. ^¶^ Computed using unrounded values. mg/dL= milligrams per deciliter. $a_{RC}$ = cell frequency for respective row (R) and column (C). Cov= covariance. Var = variance. ${\hat{RR}}_{G}$ = relative risk estimate for indicated group (G). $P_{Int}=$ *p*-value for interaction. RLD = relative risk-reduction on the log-difference scale. ${\hat{\zeta }}_{RRR}$ = test statistic for the ratio of relative risks.

**Table 2:** Exact statistics simulated from 10,000,000 multinomial observations.

Characteristic	Simulated Exact Value
$Var\left[log\left({\hat{RR}}_{1}\right)\right]$	0.01355
$Var\left[log\left({\hat{RR}}_{2}\right)\right]$	0.00622
$Cov\left[log\left(\hat{{RR}_{1}}\right),log\left(\hat{{RR}_{2}}\right)\right]$	0.00192
${\hat{\zeta }}_{RRR}$	2.2784
$P_{Int}$	0.01566

Var = variance. ${\hat{RR}}_{G}$ = relative risk estimate for indicated group (G). ${\hat{\zeta }}_{RRR}$ = test statistic for the ratio of relative risks. $P_{Int}=$ *p*-value for interaction.

## Data Availability

Data are contained within the article.

## Abbreviations

The following abbreviations are used in this manuscript:

ASCVD	Atherosclerotic cardiovascular disease
Cov	Covariance
EM	Expectation–maximization
GLM	Generalized linear model
LDL-C	Low-density lipoprotein
Log	Logarithm
OR	Odds ratio
RR	Relative risk
RRR	Ratio of relative risks
Var	Variance

## Appendix A. SAS GENMOD Code and Output for Validating the Manual Results of Example 1

**SAS Code**
option ls=180; data a; input group $ count response; cards; T0 54 1 T0 6 0 T1 48 1 T1 12 0 T2 36 1 T2 24 0 ; proc genmod data=a descending; class group; model response=group/dist=bin link=log; weight count; estimate “RR1” group 1 0 −1 / exp; estimate “RR2” group 1 −1 0 / exp; estimate “RRR” group 0 1 −1 / exp;

**Output (screenshot from the SAS system)**
